# Determining Risk Factors of Bleeding in Patients on Warfarin Treatment

**DOI:** 10.1155/2014/369084

**Published:** 2014-11-09

**Authors:** Evren Uygungül, Cuneyt Ayrik, Huseyin Narci, Semra Erdoğan, İbrahim Toker, Filiz Demir, Ulas Karaaslan

**Affiliations:** ^1^Department of Emergency Medicine, Silifke State Hospital, Mersin, Turkey; ^2^Department of Emergency Medicine, Faculty of Medicine, Mersin University, Mersin, Turkey; ^3^Department of Biostatistics, Faculty of Medicine, Mersin University, Mersin, Turkey; ^4^Department of Emergency Medicine, Tepecik Research Hospital, İzmir, Turkey; ^5^Department of Emergency Medicine, State Hospital, Niğde, Turkey; ^6^Department of Emergency Medicine, State Hospital, Balıkesir, Turkey

## Abstract

*Background*. Warfarin is a commonly used oral anticoagulant agent. The most common adverse effects of warfarin are bleeding complications. *Methods*. We performed a 1-year retrospective chart review of emergency department patients using warfarin. A total of 65 patients with bleeding disorder (study group) and 63 patients without bleeding (control group) were included, making up a total of 128 subjects. Demographic data, frequency of international normalized ratio (INR) checks, and routine blood results were extracted. Logistic regression analysis was used to determine which factors were most closely associated with bleeding complications. *Results*. Median age was 62.0 ± 14.4 and 61.9 ± 14.5 for study group and control group, respectively. Educational status and frequency of INR checks were similar in both groups (*P* = 0.101 and *P* = 0.483, resp.). INR levels were higher in the study group (5.45 ± 3.98 versus 2.63 ± 1.71, *P* < 0.001). Creatinine levels were also higher in the study group (1.14 ± 0.57 mg/dL versus 0.94 ± 0.38 mg/dL, *P* = 0.042). Acetylsalicylic acid use was more frequent in the study group and was associated with a 9-fold increase in bleeding complications (*P* < 0.001). *Conclusions*. High INR levels, high creatinine levels, and acetylsalicylic acid use were associated with bleeding complications in ED patients using warfarin.

## 1. Introduction

The oral anticoagulant agent warfarin is used for treatment and prophylaxis of various thromboembolic diseases such as deep vein thrombosis, pulmonary embolism, stroke, heart valve replacement, and atrial fibrillation [1]. The most common parameter used to monitor its effect on the clotting system during follow-up of patients is the international normalized ratio (INR). Usually, the dose of warfarin is frequently adjusted to maintain the INR level between 2 and 3.5 based on the underlying disease. Because of its narrow therapeutic index, patients using warfarin may have minor and major bleeding, especially in those with poor medication compliance [2, 3].

Patient-related risk factors for bleeding while using warfarin include age, INR, creatinine level, genetic characteristics (*VKORC1* and* CYP2C9* mutations), duration of warfarin use, and concomitant acetylsalicylic acid use. Conflicting results were attained from studies assessing the relationship between age and bleeding [4, 5]. High INR levels are an important risk factor for bleeding [6]. Renal failure and concomitant use of warfarin and acetylsalicylic acid are also risk factors for bleeding [7, 8]. The associations of bleeding with chronic diseases, liver function, and infectious diseases are still not well defined.

In this study, we aimed to determine the risk factors for bleeding in our emergency department (ED) in patients taking warfarin.

## 2. Materials and Methods

A 1-year retrospective charts of adult patients (age ≥ 17 years) presenting to the emergency department of our tertiary care university hospital were examined. Charts of those taking warfarin were then examined further to extract demographic and clinical data for analysis. Patients were divided into two groups: study group (SG) consisting of 65 patients on warfarin use who were admitted to our emergency service for major or minor bleeding episodes and control group (CG) consisting of 63 patients on warfarin treatment who were admitted to our emergency service for various reasons without bleeding.

Patients sent to our ED from another healthcare facility for purposes of vitamin K, plasma, or blood administration were excluded from the analysis.

Demographic data such as age, gender, educational status, INR control intervals, and blood analysis data such as INR, hemoglobin, platelet count, AST, ALT, creatinine, and CRP levels were recorded into questionnaire. Lab values: hemoglobin (nl 11.7–16 g/dL), platelet count (nl 150–400 × 10^3^/*μ*L), CRP (nl 0–5 mg/dL), creatinine (nl 0–0.9 mg/dL), AST (nl 0–32 U/L), ALT (nl 0–55 U/L), and INR. Hemoglobin and platelet count were determined using an electronic cell counter (Sysmex XT 2000). Serum AST and ALT levels were measured by enzymatic kinetic methods (COBAS INTEGRA 800). Serum creatinine levels were measured by enzymatic calorimetric methods (COBAS INTEGRA 800). Serum CRP levels were measured by turbidimetric methods (COBAS INTEGRA 800).

## 3. Statistical Methods

SPSS version 11.5 (statistical package for the social sciences windows) was used for statistical analysis. The Shapiro-Wilk test was used to determine if the continuous variables had a normal distribution. Student's* t*-test was used to assess the difference of mean age values and Mann-Whitney* U* test was used to assess the difference of biochemical parameters such as INR, AST, ALT, drug dose, and duration of drug use. Pearson chi-square and likelihood chi-square tests were used to assess differences between categorical variables. Descriptive statistics (minimum, maximum, mean, standard deviation, and median and 25th–75th quartiles) for continuous variables and the number and percentages for categorical variables are given. Logistic regression analysis was performed to evaluate risk factors. Comparisons with a *P* value of less than 0.05 were considered statistically significant.

## 4. Results

During the study period, 128 patients who presented to the ED using warfarin were included. Indications for warfarin use were deep vein thrombosis (7%), pulmonary embolism (8%), atrial fibrillation (25%), prosthetic heart valve (38%), cerebrovascular disease prophylaxis (20%), and coronary artery bypass surgery (2%). Of these patients, 65 were determined to have bleeding as a cause of presentation to the ED (SG), and 63 patients had no bleeding (CG).

Comparisons between the two groups (SG and CG) regarding age, gender, level of education, and acetylsalicylic acid use are listed in Table 1. While differences in age (mean age of 62 years in both groups) and gender were not different between groups, acetylsalicylic acid use was much more common (61%) in the study group compared to the control group (14%, *P* < 0.001). The level of education was lower in the study group, but this comparison did not reach statistical significance (*P* = 0.101).

Comparison between the two groups, regarding warfarin dose, duration of warfarin use, INR sampling frequency, and laboratory results is displayed in Table 2.

Warfarin dose, duration of warfarin use, and platelet counts were not significantly different in the two groups (*P* = 0.53, *P* = 0.58, and *P* = 0.23, resp.). Mean INR levels were significantly higher in SG (5.45 ± 3.98) than in CG (2.63 ± 1.71) (*P* < 0.001). Mean CRP levels were significantly higher in SG (39.8 ± 66.3 mg/dL) than in CG (15.5 ± 38.4 mg/dL) (*P* = 0.002).

Mean creatinine and BUN levels were also significantly higher in the SG patients (*P* = 0.042 and *P* = 0.009, resp.).

When significant parameters such as INR, hemoglobin, creatinine, BUN, CRP, and concomitant use of acetylsalicylic acid were examined by logistic regression analysis, INR (1.42-fold increase) and concomitant acetylsalicylic acid use (9.25-fold increase) were significantly associated with a higher risk of bleeding. Odds ratios with confidence intervals for various parameters are listed in Table 3.

## 5. Discussion

The oral anticoagulant warfarin is commonly used in the prophylaxis and treatment of several thromboembolic diseases. It is also ranked among the medications with the highest adverse effects due to its narrow therapeutic index, variation in effectiveness with dietary changes, and noncompliance with INR monitoring. Minor and major bleeding episodes (sometimes fatal) are not uncommon.

Regarding the level of education in patients with bleeding-related ED visits, 83% of our SG patients had primary school level education or less. This very high percentage should lead clinicians to strongly consider giving more education about warfarin's risks and arranging closer follow-up of patients with little education.

In the studies of warfarin users that assessed the relationship of age to bleeding episodes, the risk was lower in younger patients, especially those who had stable INR levels over the long term [3]. In these patients, the authors suggested extending the duration between INR checks from every 3-4 weeks to 8–12 weeks. In a study of 102 cardiac patients on warfarin, INR levels were higher in older patients compared to younger patients taking the same warfarin dose [4]. The authors concluded that age is an important risk factor for bleeding in patients using warfarin. While Fang et al. found increased bleeding rates (including intracranial hemorrhage) in patients over 80 years, in their study of over 13,000 atrial fibrillation patients, these rates were similar in those taking and not taking warfarin [5]. Concomitant acetylsalicylic acid use was not an important contributor to bleeding risk in their study. They concluded that warfarin is reasonably safe to use in elderly atrial fibrillation patients if they are carefully monitored. Our patients on warfarin who presented with bleeding to the ED were not significantly older than those who had no bleeding complaint.

Other factors that might influence the risk of bleeding are frequency of INR checks, dose of warfarin, and genetic variants. Most of our patients had regular INR check-ups, every 30–90 days; the intervals in both groups were not significantly different from each other. The* VKORC1* and* CYP2C9* gene mutations are associated with higher bleeding risk in patients on warfarin, thus dosages should be modified when these mutations are found [7, 8].

Without careful monitoring, bleeding risks increase with the duration of warfarin use. Hylek et al. found that bleeding risk increased significantly in patients over 80 years who had an INR level over 4 and who had been using warfarin for more than 90 days [9]. Similarly, in a study of 184 warfarin-using patients younger than 12 years, INR levels were higher in those using warfarin for an extended duration [10]. However, Aspinall et al. found that the duration of warfarin use was not an independent risk factor for increased bleeding risk in patients followed up in an anticoagulation clinic [11].

Those with moderate to severe renal failure on warfarin have higher INR levels and risk of bleeding compared to those with normal kidney function [12, 13]. We found that our patients using warfarin who presented due to bleeding (SG) had higher creatinine levels than CG patients. Careful adjustment of warfarin dose and frequent monitoring of INR may help prevent bleeding episodes in these patients.

Many drugs affect the metabolism of warfarin, increasing or decreasing its levels and activity [1, 14]. Acetylsalicylic acid is probably the most problematic agent, as it is commonly used and its platelet-inhibiting properties act synergistically with those of warfarin to enhance any bleeding that might occur. The risk of clinically important minor and major bleeding increases 1.5–2-fold when acetylsalicylic acid and warfarin are used together [15]. As seen in Table 3, the use of aspirin was much more frequent in our patients with bleeding than in those without bleeding complaints.

Gando et al. found that coagulation pathways were triggered in cases of severe infections; therefore, one might expect an increased bleeding risk in patients using warfarin who have severe infections [16]. In our study, we found higher CRP levels in our SG patients than in our CG patients. However, most patients had no symptoms or signs of clinical infection; thus, it is difficult to associate the higher CRP levels with a specific infection or inflammatory response. Further studies should be done to clarify this relationship.

## 6. Limitations

This was a small, retrospective study. Bleeding scores and creatinine clearance were not calculated. To increase the success rate of data prediction, it was aimed to reach at least 30% of patients on warfarin treatment who were admitted to emergency service. After analyzing computer data retrospectively, 50% of patients on warfarin use were considered as reachable.

## 7. Conclusion

In emergency department patients using warfarin, increased INR level, high creatinine, and concomitant use of acetylsalicylic acid are strongly associated with a bleeding-related visit. These results will be useful in our practice here, as this is the first study in Turkey of warfarin-using patients in the emergency department and their clinical and laboratory findings. In the future, studies of genetic variations among emergency department patients using warfarin who have bleeding-related complaints should be performed.

## Figures and Tables

**Table 1:** Demographic data (age, gender, and education level), concomitant use of acetylsalicylic acid, and INR sampling frequency in 128 patients using warfarin with bleeding-related (SG) and non-bleeding-related (CG) reasons for their emergency department visit.

	CG		SG		*P*
	*n*	%	*n*	%	
Gender					
Female	33	52.4	38	58.5	0.489
Male	30	47.6	27	41.5	
Highest level of education					
Unschooled	10	15.9	15	23.1	0.101
Primary school	33	52.4	39	60.0	
High school	13	20.6	4	6.2	
University	7	11.1	7	10.8	
Time between INR checks					
Unscheduled	4	6.3	11	16.9	0.483
<30 days	15	23.8	10	15.4	
30–90 days	42	66.6	43	66.2	
>90 days	2	3.3	1	1.5	
Acetylsalicylic acid use					
Yes	54	85.7	25	38.5	**<0.001**
No	9	14.3	40	61.5	

**Table 2:** Dose and duration of warfarin use and laboratory results of 128 patients using warfarin with bleeding-related (SG) and non-bleeding-related (CG) reasons for their emergency department visits.

	CG			SG			*P*
	Min–max	Mean ± SD	Median [25–75% quartiles]	Min–max	Mean ± SD	Median [25–75% quartiles]	
Age (years)	23–88	61.9 ± 14.5	—	23–91	62.0 ± 14.4	—	0.970
Dose (mg/week)	17.5–42.5	25.36 ± 8.24	22.5 [17.5–35.0]	8.75–70.0	30.17 ± 13.42	35 [17.5–35.0]	0.053
Duration (months)	1–276	49 ± 59	24 [10–72]	1–288	53 ± 60	24 [12–72]	0.583
INR	0.88–11.00	2.63 ± 1.71	2.19 [1.64–3.03]	1.08–18.30	5.45 ± 3.98	4.46 [2.38–6.70]	**<0.001**
Hemoglobin (g/dL)	7.4–16.4	12.46 ± 2.12	12.60 [10.90–14.2]	4.9–17.1	11.10 ± 2.68	11.1 [9.05–13.40]	**0.005**
Platelet count (10^3^/*μ*L)	109–489	246 ± 80	229 [188–306]	81–585	266 ± 96	247 [204–309]	0.231
AST (U/L)	10.3–71.6	29.3 ± 14.6	23.9 [19.1–36.4]	9.2–149.0	33.0 ± 24.2	25.3 [19.9–35.4]	0.710
ALT (U/L)	5.3–94.0	22.4 ± 14.5	17.8 [13.7–25.3]	3.7–136.0	23.4 ± 21.2	17.5 [14.0–23.4]	0.598
Creatinine (mg/dL)	0.5–2.7	0.9 ± 0.4	0.8 [0.7–1.1]	0.5–3.1	1.1 ± 0.6	1.0 [0.7–1.4]	**0.042**
BUN (mg/dL)	13.2–132.1	37.6 ± 21.8	32.3 [26.4–40.1]	12.9–199.2	50.3 ± 33.8	39.1 [28.7–58.3]	**0.009**
CRP (mg/dL)	0.09–271.97	15.46 ± 38.38	4 [1.5–12.8]	0.07–369.90	39.83 ± 66.31	13.50 [2.70–57.20]	**0.002**

**Table 3:** Odds ratios (ORs), 95% confidence intervals (CIs), and *P* values when comparing laboratory values and acetylsalicylic acid use between patients using warfarin with bleeding-related (SG) and non-bleeding-related (CG) reasons for their emergency department visits.

	OR [95% CI]	*P*
INR	1.417 [1.149–1.748]	**0.001**
Hemoglobin	0.812 [0.656–1.005]	0.055
Creatinine	1.041 [0.173–6.273]	0.965
BUN	0.994 [0.964–1.026]	0.722
CRP	1.008 [0.998–1.018]	0.103
Acetylsalicylic acid	9.255 [3.062–27.968]	**<0.001**
